# Our Experience and Clinical Findings in Perineal Burns: Implications for Patient Prognosis—A 3 Year Retrospective Study

**DOI:** 10.3390/medicina60122009

**Published:** 2024-12-05

**Authors:** Matei Iordache, Eliza-Maria Bordeanu-Diaconescu, Andreea Grosu-Bularda, Mihaela-Cristina Andrei, Adrian Frunza, Sabina Grama, Raducu Costache, Tiberiu-Paul Neagu, Ioan Lascar, Cristian-Sorin Hariga

**Affiliations:** 1 Department of Plastic Surgery and Reconstructive Microsurgery, “Carol Davila” University of Medicine and Pharmacy Bucharest, 010825 Bucharest, Romania; matei.iordache@umfcd.ro (M.I.);; 2 Burn Centre, Emergency Clinical Hospital of Bucharest, 014461 Bucharest, Romania

**Keywords:** burns, perineal burns, mortality, infections

## Abstract

*Background and Objectives:* Burn injury represents a very important public health problem that affects all age groups. Of all burns, of particular interest is that of the perineum. Despite the importance of the subject, unfortunately, the medical literature on this anatomical region is sparse. With this study we aim to analyze the characteristics of burns affecting the perineal area, the physiopathologic implications of this injury, the influence of patient prognosis, possible complications and therapeutic guidelines. *Materials and Methods:* This study is formed by a retrospective analysis of cases that were admitted over a period spanning 3 years with a total of 258 burned patients. After inclusion criteria, we selected 49 patients who had perineal burns and compared this group to a non-perineal burns lot of 198 patients (11 were excluded). We studied their characteristics and the demographical aspects that we deemed most important to their condition: age, sex, burn percentage of total body surface area (TBSA), the percentage of third-degree lesions, comorbidities, and associated infections, inhalation injuries and we calculated the significant scores such as the Abbreviated Burn Severity Index score (ABSI). *Results:* The patients in our study mostly had severe extensive burns (64.9% mean TBSA) which were also underlined by the mean ABSI of 10.88 ± 2.46 thus having a poor prognosis considering their age, the percentage of burned area, the presence of third-degree burns and inhalation injuries. In our study, perineal burns were usually associated with burns of adjacent regions abdominal wall burns comprising 51% and thigh burns comprising 97.9% of the associated injuries. This relationship both explains their presence in mostly severe cases with higher TBSA and also underlines the issues that derive from the burns of the perineum and their several complications which lead to an unbalance of the patients. The treatment of perineal burns still remains much debated in the literature when considering their indications and can become rather complex in the sequelae setting. *Conclusions:* The issue of burns remains one of the most important subjects in plastic surgery. Being a region hard to treat but with a big influence on patient evolution and survival chances prevention remains a key factor.

## 1. Introduction

Burns are a major global public health issue that affects individuals of all ages. Whether caused by flames, scalds, chemical exposure, or electrocution, burn injuries occur more frequently in developing or low-income countries, where there is often insufficient awareness about burn prevention measures [1,2,3]. The severity of this problem stems not only from the high incidence—over 300,000 deaths caused by burn injuries—but also from the prolonged hospitalization, increased mortality, as well as the psychological and emotional challenges that arise and the potential long-term complications or sequelae [4,5,6,7]. The injury leads to the onset of a severe systemic inflammatory response with a hypermetabolic reaction, leading to septic complications, multiple organ failure and an increased rate of death [8,9].

The American Burn Association (ABA) has established guidelines for burn patient referral, highlighting the importance of burns in functional areas, particularly the perineum, due to their significant impact on quality of life, morbidity, and mortality [10,11]. ABA has classified perineal burns as being major, and several single-institution studies have estimated their prevalence between 1.7–13% of the total burn hospital admissions [12]. In most cases, perineal burns are observed in patients with a larger total body surface area (TBSA) affected. Although the perineum is typically a protected region and represents only 1% of the TBSA, studies have found that patients with perineal burns have an average of 21% to 56% TBSA affected. Perineal burns tend to have a poor prognosis due to the association with urinary tract infections, increased incidence of hospital-acquired infections and bacteremia, and higher mortality [12,13].

Since genital area burns are uncommon, there is unfortunately a lack of guidelines in the literature, providing only limited information on the topic [14]. Our study presents a comprehensive overview of current data and treatment approaches for perineal burns, while also comparing the similarities and differences with our own experiences, studying the impact of the problem on survival chances and morbidity. We aimed to analyze the characteristics of perineal burns, the implications of this injury and the influence on the prognosis while establishing also the therapeutic protocols.

In formulating an adequate strategy for the difficult management of this particular anatomical region, we found it important to depict as well the degrees of burn lesions as well as the means of treatment.

## 2. Materials and Methods

We performed a retrospective study including the 258 patients admitted to the Critical Care Burn Unit of the Clinical Emergency Hospital Bucharest, from 1 June 2016 until 1 June 2019. Of the total number of patients admitted to the hospital, we studied those who met the inclusion criteria: age ≥20 years and the presence of perineal burns. We excluded the patients who were ≥91 years old and those transferred to another unit.

The following demographic and clinical characteristics of the included patients were obtained: age, gender, mechanism of injury, %TBSA burned, presence of third-degree burns, presence of inhalation injuries, transfer from another hospital, length of stay (LOS) and the surgical procedures performed.

We recorded the results of the microbiological assessment. Microbiological screening was conducted at admission including the following: burn wound cultures, blood cultures both aerobic and anaerobic, tracheal aspirate cultures and urinary cultures. In our burn center, we do not administer antibiotherapy routinely; we only administer antibiotic prophylaxis perioperatively. If infection is clinical and paraclinically suspected antibiotherapy is started empirically and modified afterwards according to the antibiogram results. Further on, we conducted weekly microbiological screening of the burned patients to monitor changes in the microbial flora, collecting samples from burn wound areas, including the studied areas. Aside from this protocol, microbiological testing was performed on every suspicion of developing an infection. When sepsis was suspected, an extensive panel was conducted, including a tracheal aspirate, uroculture, burn wound cultures and hemoculture.

We calculated the Abbreviated Burn Severity Index score (ABSI), which is used to predict burn mortality and we performed the statistical analysis of the variables using Microsoft Excel as well as IBM SPSS Statistics 24 to calculate the Chi-Square test which reported the χ^2^ and *p* values, comparing the patients with perineal burns with the control group considering a significant *p*-value of ≤0.05. The analysis of the aggravating factors on survival was conducted using a binary logistic regression, reporting the model fit coefficient (R^2^), the estimated value (B,SE), the significance test (Wald) and the odds ratio (OR = Exp(B)).

For this study, we received the required approval from our hospital’s ethics committee (approval number 2572/26 March 2024) and conducted our research in accordance with all principles outlined in the Declaration of Helsinki.

## 3. Results

In our study, we identified a total of 258 patients admitted for burn injuries during the three-year period, of whom 49 had burns in the perineal region (19.8%) and were further analyzed in this study, while comparing them to the remaining 198 patients who met the eligibility criteria.

The vast majority of the patients were admitted to the hospital by transfer from another hospital around the country—34 patients (69.4% of patients), while only 15 (30.6%) were directly admitted.

The characteristics of patients with perineal burns as well as the patients pertaining to the control group are depicted in Table 1.

**Figure 1:**
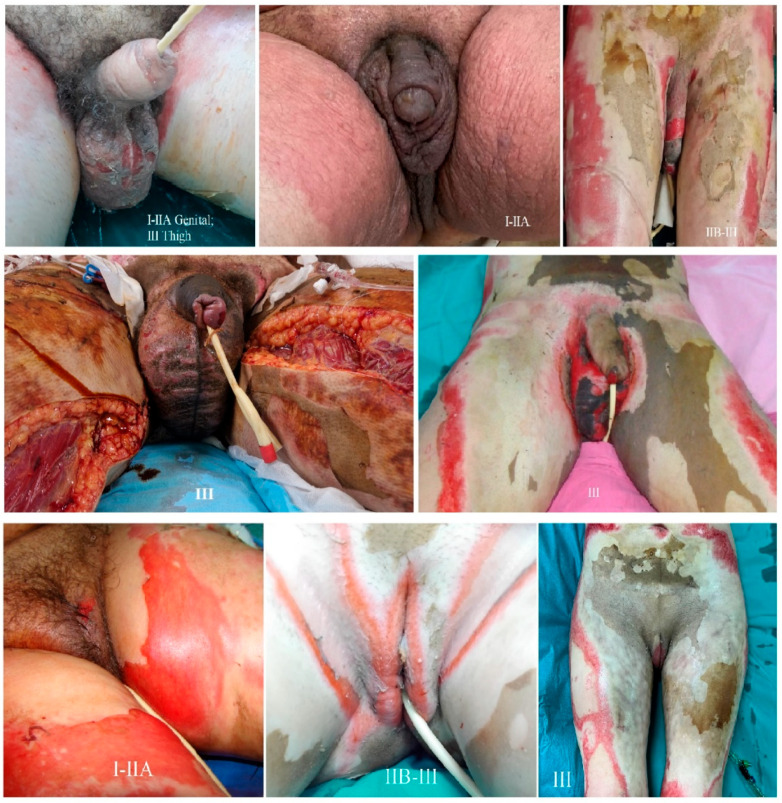
The Figure illustrates the clinical appearance of different degrees of severity of perineal burns, depending on their depth. This classification is based on the American Burn Association Criteria [10].

The depth of burns was assessed in our cases according with the American Burn Association (ABA) Classification, the specific aspects of each degree of burns being presented in Figure 1.

The mean TBSA affected by the burn injury in our 49 patients lot was 64.9%. A total of 45 patients (91.8% of patients) had third-degree burns, with the remaining 4 being evenly split between the two genders. In the case of patients with perineal involvement, the proportion of grade III is high (91.8%), a proportion that does not differ significantly based on sex (χ^2^(df = 1) = 1.57; *p* = 0.278 > 0.05). For patients without perineal involvement, the proportion of grade III was approximately 60% (62.1%), a proportion that does not differ based on sex (χ^2^(df = 1) = 0.08; *p* = 0.454 > 0.05). Inhalation lesions were identified in 34 patients (69.4% of patients).

Regarding the anatomic distribution of burns, 48 patients (97.9% of the patients) with perineal burns also associated burns of the thighs, with a single case (2.04%) of a patient with an isolated perineal burn. Another common association was with anterior abdominal burns, which were found in 25 patients (51% of patients).

The overall mortality for the patients admitted in the 3 years was 55.87%, while in the case of patients with perineal burns, the mortality reached 77.5% (38 of the total of 49 patients). The statistical analysis showed a high mortality rate due to burned TBSA, third-degree injuries and a high ABSI score.

The mean LOS in the hospital was that of 17.4 days with 35 patients being admitted for a period of time equal to or less than 14 days. For a better relevance, we calculated the mean LOS for the 10 surviving patients, being 46.9 days.

Associated comorbidities were also assessed with five patients presenting high blood pressure, four with cardiovascular diseases, five patients with type II diabetes, two obese patients, two patients with chronic obstructive pulmonary disease, one patient with cirrhosis, one case with scrotal hernia and another with inguinal hernia. One patient associated with traumatic rupture of the spleen. Neuropsychiatric conditions were found to be associated with traumatic injuries in six patients—four having chronic alcohol abuse while two had Alzheimer’s disease. Of the total number of cases, 11% had a suicidal mechanism and all of these had a burned surface area bigger than 60% TBSA.

**Figure 2:**
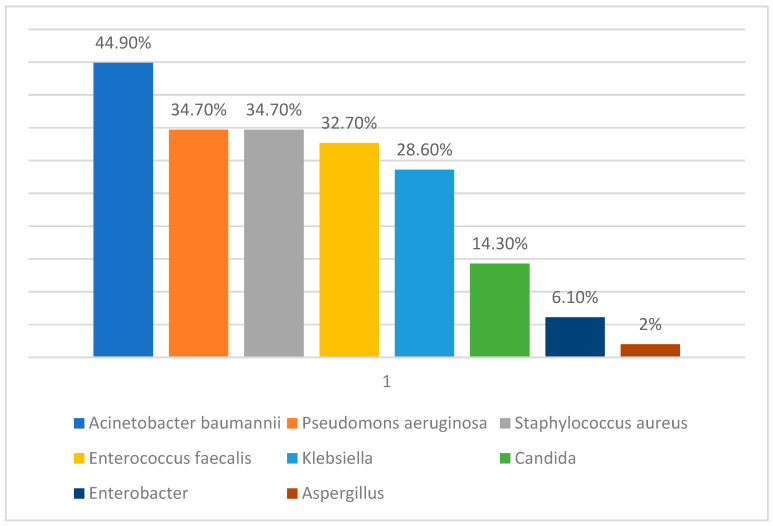
Microorganisms identified in perineal burn wounds.

**Figure 3:**
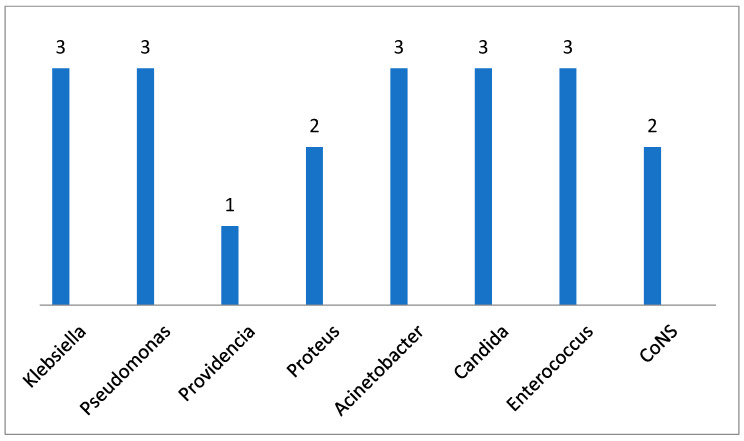
Distribution of microorganisms in the 13 patients with positive urine cultures (CoNS = Coagulase-Negative Staphylococci).

Regarding the infectious complications, both bacteria (33 patients, 67.4% of patients) and fungi (8 patients, 16.3% of patients) were identified in perineal burn wounds, part of the patients presented 3 or 4 germs in the perineal burn wounds, with the distribution shown in Figure 2. Of the total number of patients, 12 (24.5%) had positive hemoculture, while 13 (26.5%) had documented infections of the genitourinary system as shown in Figure 3.

**Figure 4:**
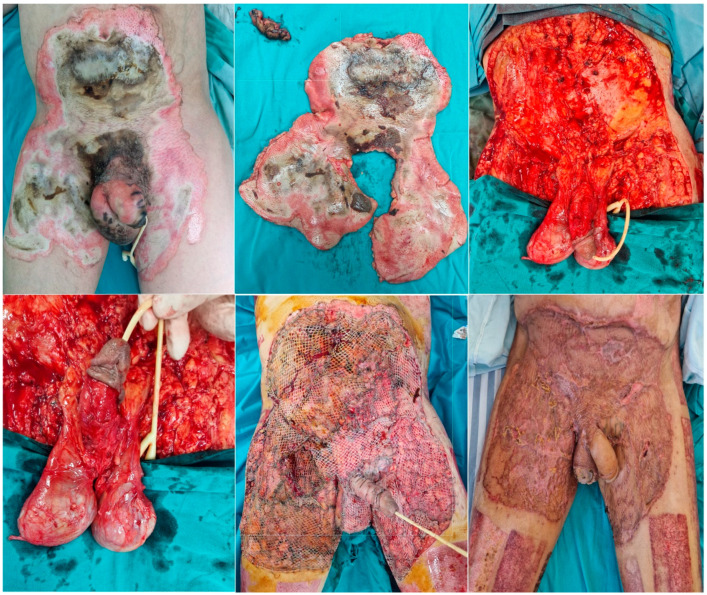
A case from our clinic with the classical association between the genital and perineal burns in the lower abdomen and the upper thigh regions. Upper left is the initial aspect with most of the burns being third-degree. Upper central is the excised segment of the necrotic tissue with the resulting defect (upper right and lower left). The last two pictures show the aspect of the grafted regions immediately postoperatively and 2 months away, respectively. Note the different aspects of the skin grafts, being thicker on the penis to prevent contractures compared to the other grafted regions.

The therapeutic principles regarding burns in the perineal region in our cases prioritize urgent urethral catheterization before significant edema sets in. Urethral lesions were ruled out after performing an urologic evaluation. The patients benefited from general hygiene, lavage with antiseptic solutions, and non-excisional debridement of the burn lesions. Subsequently, the lesions were dressed daily with antiseptic and antimicrobial ointments, depending on the depth of the injury. The assessment of burn depth was performed through dynamic examination over the following days, as this is a well-vascularized anatomical area with functional impact, and it is important to define the extent of the lesions before performing excisional debridement. Surgical intervention was guided by the severity and depth of the burns. Superficial burns were managed conservatively with topical agents and thorough cleaning, as they have the potential for spontaneous healing. Partial-thickness burns required excision of necrotic tissue and skin grafting in the cases where healing was delayed beyond two weeks or if granulation tissue did not develop adequately. Full-thickness burns, necessitated early eschar excision to reduce infection risk and prepare the wound bed for grafting. Hygiene of the region is mandatory to prevent fecal contamination, and, in two cases, a fecal collection system was installed. In the case of third-degree burns, the approach is to initiate prompt surgical treatment, which includes excision and grafting of the lesions; preferably, non-expanded grafts are used if donor sites are available. Out of the 49 studied patients 39 required surgical treatment including emergency escharotomies and sequential excision and grafting of the third-degree burns. Aside from these, two patients also required amputations of the lower limbs—one bilateral and the other one unilateral thigh amputation and five patients had to be tracheostomized. Out of the 10 surviving patients, 7 cases required partial grafting in the inguino-genital region and 1 case (illustrated in Figure 4), required extensive grafting of the area. One patient required partial regrafting of the scrotal area due to not adhering to the posturing recommendations and graft lysis. In two cases, the lesions were partial-thickness burns that healed conservatively. Healing was uneventful in these cases, with no complaints regarding urinary function.

## 4. Discussions

Burn injury represents a significant public health burden affecting individuals of all ages [1]. Among all types of burns, those affecting the perineal region are among the rarest, and unfortunately, there is limited information in the literature regarding treatment guidelines [14].

We observed a notably high incidence of perineal burns in our center, compared to what we found in the literature from burn units worldwide. While most of the studies found in the literature report an incidence of perineal burns ranging from 1.7% to 13% [15], with, 10.1% in another article [16], our study has a significantly higher frequency of such burns—19.8%. The chi-square test comparing the proportion obtained for perineal burns in our study with the maximum proportion reported in the literature indicates that this difference is statistically significant (χ^2^(df = 1) = 5.24; *p* = 0.022 < 0.05). A potential explanation for this would be that we admitted more severely burned patients in our center, with higher TBSA and deeper burn lesions, since we are the only accredited burn center in the country, leading to the selection of the most severe cases to our unit. Out of 49 patients, 34 were admitted by transfer from another hospital in the country, while only 15 were directly admitted, making us a referral center for the most severe of burns.

Although the TBSA of the genitalia is only 1% [17] and the entire perineum comprises 4–6%, the American Burn Association and other working groups [16] classify these as major burns due to their significant anatomical and functional importance [12,18]. The genital area is well protected because of the anatomical location and the characteristics of the skin of the penis and the scrotum with the cremasteric reflex. As such, they are rarely isolated injuries, in our study we found correlations with other burned regions that significantly contributed to an increase in the total burned surface area [14]. Patients with a higher burned TBSA were, therefore, at an increased risk of complications. Out of the total of 49 burned patients, the most frequent association is that with burns of the thigh region, appearing with an extremely high rate—48 of the cases—97.9%. This generates a unique situation that may influence patient outcomes by increasing the risk of developing deep venous thrombosis [19], which could potentially lead to pulmonary embolism. In many cases, this is further derived from the challenges of mobilizing the patients [16,20]. The second most common association is that of burns in the abdominal region which appear in 25 of the cases—51%. Thus, it becomes pretty evident that isolated perineal burns are a rarity, in our case study having observed only one case of isolated burns of the perineal region. In a study, Sajad et al. [18] also reported 49 patients with perineal burns, discovering the same frequent anatomical associations but managing only the contractures that were reported on for a longer period of time—15 years.

Regarding the severity of burns in our patients, we found that the mean surface of TBSA burned in our study was very high—64.9% compared to 38.4% in other studies [16]. Most of the patients presented third-degree burns as well—91.8%, with significantly deeper burns of the perineal region than those reported in the literature. Previous studies mostly describe superficial partial thickness injuries of the perineum [16,21,22].

When referring to the sex ratio, the vast majority of our patients were males—36, representing 73.5%, with the females being only 13—26.5%, comparable to data from the other studies [12,16]. The studies already published in the literature hypothesize the fact that male patients are more commonly affected [20,23], likely because they are at a higher risk of engaging in activities that could result in burns to this area, having more dangerous jobs [1] and added to this is the fact that there is a significant difference in the anatomy of the external genital area. The penis as well as the scrotum represent a bigger surface that could be affected, while not being as well protected by the thigh folds as in the case of the female patients [12]. A particular instance is that of war trauma which usually has a homogenous cohort of patients, young healthy males [24]. This type of patient allows us to underline the importance of the role of perineal burns to the overall risk of the patient, due to the fact that a higher mortality rate, increased hospital stay and more severe risk of infection (by flora contamination and urinary tract infections) were observed, excluding the additional risks that may occur in the cases of civilian patients who may have pre-existing conditions [24].

Regarding the age distribution, we observed two peaks with a higher perineal burn incidence represented by the decade 41–50 years (similar to the data reported by Li H et al. [25]), another peak being in the age decade between 71 and 80 years, comprising our largest group, while the rest were somewhat evenly distributed between the other age intervals. The patients included in our study were all 20 years or above and we did not see a gradual increase in incidence as age advances with a peak only at older ages (71–80 or 80+ ranges) [26,27]. Hence, perineal burns represent a social problem as they affect more often the active working patients, being a severity factor for them, or the elderly patients by worsening their prognosis, around half of our patients being 60 years of age or above, this age being known as a severity factor in the evolution of the patients [28].

Psychiatric disorders are frequently encountered in our burned patients. Out of the total 49 patients with perineal burns, 11 cases were with suicidal attempts, representing 22.5% of the total cases. The ratio of suicidal patients is similar to the ones found in the literature since they report 23.8% among adults and, similar to their findings, all of our patients had more than 60% TBSA [29]. Their lesions were severe, with 2 of the patients having 60% and 4 with more than 90% TBSA burned [16]. This particular group of patients is important since the suicidal attempt usually leads to more severe traumas [30]. Pre-existing psychiatric illness has been linked to worsening the vital prognosis, reduced compliance, a greater number of procedures required with inadequate physical recovery, higher infection risk, an increased LOS and poorer overall treatment adherence [31].

Patients included in our study had severe burns, illustrated by the high incidence of inhalation injuries in our patients, appearing in 34 of the total 49 cases which represents 69.4%. The presence of inhalation injury is a negative prognostic factor for the evolution of the patient, as there have frequently been observed cases that associate head and neck lesions with inhalation burns which occur together with burns of the perineal region in the adult population [16].

While performing a statistical analysis of our patients, we can confirm these data as the most important factors that we found to be carrying a higher vital risk—age, the presence and percentage of third-degree burns and the high ABSI rates. Age is a significant positive predictor (Wald(df = 1) = 3.68; *p* = 0.055), with a predictive capacity ranging between 8.5% and 13.9% of cases. The positive value of the estimate (B = 1.66 and SE = 0.86) indicates an odds ratio of 5.3 (Exp(B) = 5.25; 95% CI: 0.97–28.6), meaning that patients over 60 years of age have 5.3 times higher odds of dying compared to those under 60 years old. The severity of the burn is a marginal positive predictor (Wald(df = 1) = 5.78; *p* = 0.016), with a predictive capacity ranging between 13.1% and 21.4% of cases. The positive value of the estimate (B = 2.97 and SE = 1.24) indicates an odds ratio of 19.5 (Exp(B) = 19.5; 95% CI: 1.73–219.49), meaning that patients with third-degree burns have 19.5 times higher odds of dying compared to those with lower levels of injury.

In order to assess the patient prognosis we used the aforementioned data and calculated the ABSI score, which is multifactorial and extremely relevant in studying the mortality of the burned patient. The results that we obtained were high, with a mean value of 10.88 ± 2.46, with 27 patients having a score of 12 or above, the highest one being 17. This represents an indicator of high gravity, the guidelines of this score consider an ABSI of 12 or above as being of maximum threat to life with a ≤10% probability of survival [32]. ABSI is a significant positive predictor (Wald(df = 1) = 8.93; *p* = 0.003), with a predictive capacity ranging between 28.4% and 46.2% of cases. The positive value of the estimate (B = 0.82 and SE = 0.27) indicates an increase of 2.26 times (Exp(B) = 2.26; 95% CI: 1.33–3.87) in odds for each one-unit increase in ABSI.

All this evidence corroborates with the high mortality rate registered in our case series. The total lot of patients that we had initially studied in the time frame exhibited a significant average mortality of 55.87% compared to the 49 patients with perineal burns where we found a significantly higher mortality rate of 77.55%. The current literature also reports high mortality rates, with the National Burn Repository analysis proving that independently just the burns of the genital area lead to a 54% increase in overall mortality [15]. Two conclusions could be derived from this—on one hand, the fact that perineal burns appear in more severe cases with higher burned surfaces and deeper wounds, and on the other hand the fact that they bring more complications to the burned patients, significantly raising the mortality rate.

In the past 70 years, there has been registered important progress in the outcomes and the quality of care for patients with burns. Since we have an improvement in mortality rates, another marker was proposed to express the quality of care of the population and that is the LOS which is approximately 1 day per 1% of burned surface [24,33]. Its importance is because of the several possible impacts that it has, including an indicator for morbidity and other adverse events, also signifying indirectly the measure of quality of care [34]. A prolonged hospital stay would be greater than 14 days and for each 10% additional burned body surface, the LOS would increase 9 times. Aside from age and TBSA which strongly predict LOS, it seems that another important contribution is that of the perineal burns [35,36], which lead to more frequent hospitalizations and longer stays [37,38]. They are hard to manage due to the higher risk of contamination, the rates of infection are higher with loss of skin grafts and the additional stress caused by movement with the unusual contours in the area all add up to the higher LOS for perineal burns [33]. In our study group, the mean LOS was 46.9 days for the 10 surviving patients; the deceased were excluded here while they had significantly lower LOS due to the high mortality, death occurring rapidly due to burn shock and due to associated multiple organ dysfunction in this cohort of patients with extensive burns.

Infections in perineal burns are a key subject because infections are almost always inevitable in this type of burns [39], with studies demonstrating a threefold higher infection rate [40,41]. The primary sources in these patients are skin (mostly by maceration, strain or contamination), stool and urine [41]. In our burn center we have a standardized protocol for prevention and diagnosis. At admission we make a bacteriological and a fungal mapping of the patient’s status with subsequent reassessment. We identified 3–4 bacteria in 33 of our patients, 8 had fungal infections, 13 (26.5%) developed urinary tract infections and 12 (24.5%) developed sepsis.

In terms of infection or contamination of high importance is the type of bacteria. We were able to discover a lot of resistant bacteria, most of which were “ESKAPE pathogens” [42]. This acronym was developed by the Infectious Disease Society of America which managed to determine six bacterial species that are included here: Enterococcus faecalis, Staphylococcus aureus, Klebsiella pneumoniae, Acinetobacter baumannii, Pseudomonas aeruginosa and Enterobacter spp. The severity that derives from these pathogens comes in the form of a therapeutic challenge because they often develop antibiotic resistance which is difficult to treat even when using last-line antibiotics [43,44]. In our lot, we were able to find at least one ESKAPE bacteria present in 84% of the patients, in most cases being present at the time of admission which could lead to an increase in the mortality of the patient while being an important correlation factor to the evolution of perineal burned patients [45]. Studies show that 100% of the patients who had an infection and 80% of those with sepsis, had poor or average graft take. Of these, Pseudomonas aeruginosa is described as important for bacterial lysis. As such, the use of topicals to prevent the development of these bacteria becomes essential [46]. Ghasemian et al. conducted a study following the molecular characterization of Pseudomonas aeruginosa isolates from burn wounds, focusing on advanced diagnostics for antibiotic resistance, biofilm formation and virulence determinants. Innovative techniques, including phenotypic and genotypic assays, revealed significant carbapenem resistance (40%) and strong biofilm production in most isolates, complicating treatment strategies. Molecular approaches, such as PCR for resistance genes and ERIC-PCR for genetic typing, highlighted the coexistence of multidrug resistance and virulence factors. These diagnostic advancements, when available, provide important data regarding the pathogenic mechanisms and bacterial resistance profile, aiding in tailored therapeutic interventions for burn-related infections [47].

A crucial topic that warrants discussion is the precise treatment of this anatomical area, as it can elicit a significant response in the body and pose a high risk of complications. In terms of general treatment, the literature does not recommend administering routinely antibiotics in all cases of burn injuries [48]. This treatment is best reserved for infections of the burn lesions or significant bacteriuria, but always following the antibiogram [37].

When considering the local treatment, most recommendations in the literature are those of a conservative attitude toward genital burns. Harpole et al. [41] reported in a retrospective study performed on 71,895 burn patients out of which only 1245 patients had perineal burns the fact that the surgical treatment was infrequent (10.4% of cases), being mostly reserved for the male patients that had a third-degree burn, usually in means of sharp debridement and skin grafting [14]. According to the literature, wound dressings should be changed regularly based on the degree of the burn. In cases of more superficial burns the recommendations are unanimous to disinfect the wounds by using chlorhexidine or another antiseptic and then applying antibiotic pomade which may contain neomycin and bacitracin. For more profound burns, such as the third degree, there is the recommendation to use silver sulphadiazine or zinc oxide [49] to help eliminate the lost tissue and have a bacteriostatic effect [50].

However, in our study most of the patients had third-degree burns, thus the gold standard in our clinic became that of excision and skin grafting, leaving the conservative treatment for the more superficial burns which could rely on the good vascularization of this region and its high capabilities of epithelization. The postop follow-up of these patients becomes demanding because this anatomical region is irregular, highly mobile and in close proximity to the anatomical orifices [51]. Often we had to rely as well on urethral catheterization and collaborating with the urologist, but this opened the door to applying negative pressure therapy which increased the graft take and improved the healing process of these severe cases [52,53]. In particular cases with extensive, multilayered defects resulting from deep burns of the genital and perineal area complex reconstructive procedures are required using different types of flaps. Pedicled flaps are frequently used in this area if they are not affected by the injury such as inguino-pudendal flaps, fasciocutaneous thigh flaps, gracilis flaps or various free flaps [14].

A much-discussed issue is that of the necessity of urethral catheterization. Of course, this procedure bears the risk of colonization of the urinary tract and harming the urethra and bowel [37,54,55]. The infectious risk increases by 4% a day and is mainly seen with Pseudomonas aeruginosa or Klebsiella pneumoniae [38,56]. This should be put in balance with the fact that having a contaminated burn of the perineum or of the genital area could lead to the deepening of the burns and further necrosis. If need be placed, the catheter is removed as soon as the patient can spontaneously urinate and does not need anymore fluid resuscitation. In the few cases of circumferential burns with severe edema or severe cases of genital burns, there is the option of placing a suprapubic catheter [37].

When it comes to managing fecal soiling, there have been several options proposed for treating this issue such as artificial constipation or fecal management devices, or even the creation of a colostomy [16,57]. It remains a much-debated issue since there are not enough substantial studies or clinical trials that can support these treatment options [15,58]. Several reports describe diversion colostomies as being outdated and ineffective in terms of promoting wound healing [59]. Nevertheless, there are case series in the literature, such as Öhlbauer and Wallner which have reported that preventing infection by intestinal pathogens can be completed by placing rectal tubes that can be left for up to 30 days [60]. Diversion colostomies, on the other hand, are mostly used in deep perianal burns [47], and they aid in faster healing of the perineal region by reducing the contamination of the wounds and by giving access to the usage of negative-pressure wound therapy which is favorable for promoting epithelization and a higher graft take in cases which may require surgery [52].

The sequelae that appear in the long term after deep burns in this region can lead to contractures [61] which may limit smooth locomotion and may also affect sexual function and body hygiene [55,62]. There are some differences between sexes because intrinsic contractures that can appear in male patients are uncommon because of the laxity and extensibility which are characteristic of the penile and preputial skin [63]. In the cases of long-term issues, there have been described urethral stenosis, slough and hypospadias [17].

In the normal evolution of the wound healing process, several anatomical changes may occur which can lead to specific dysfunction which means psychological problems due to the distortion of the external genitalia [62,64]. A fundamental aspect of an individual’s life is that of his or her sexuality, normal genital function, and psychological issues such as self-esteem, the feeling of intimacy, and adequate sexual function [16]. In this issue, more affected are the female patients, with studies, although sparse yet, showing a significant relationship that there is between sexual satisfaction and the quality of life of women who have suffered from severe burns [65]. Since burn complications have led to sexual dissatisfaction there are studies [66] which demonstrate that 82% of the female patients with severe burns described not being happy with their sexual satisfaction level.

The limitations of this study derive from the relatively recent inauguration of this burn unit. The present study depicts the first three years of activity of the center, being the first accredited specialized unit in our country and explains the number of patients. Independent findings regarding perineal burns were difficult to assess as the large majority of these lesions were associated with other anatomical region burns, many of them being in the context of very extensive burns. In this situation, specific treatment addressed all of the lesions, making it difficult to evaluate the patient outcome strictly related to perineal area burns treatment. Another limitation is the impossibility of establishing a standardized follow-up protocol for these cases as many of them are living a long distance away from our burn unit. A future approach should focus on improving patients’ addressability in order to allow an optimal follow-up and the opportunity to address the sequelae complication in this anatomic area. A useful approach would be an elaboration of a standardized therapeutic algorithm for perineal burns in adult patients in a similar manner that Erlichman et al. provided in pediatric patients, following the perineal burns starting with the acute stage to the reconstructive procedure required, noting the patients’ outcome [67].

## 5. Conclusions

Burns continue to be one of the most significant topics in plastic surgery. Among these, perineal burns are one of the least explored areas, yet they hold considerable importance due to their significant impact on mortality. The diagnosis of perineal burns is associated with severity in cases of extensive burn trauma, indicating a likely negative outcome despite the implementation of all necessary interventions. Being an anatomical region that is difficult to treat both surgically and in terms of associated infectious complications, prevention of perineal burns remains key, and further techniques to reduce sequelae should be studied, with a strong focus on addressing infectious problems and the challenges associated with surgically covering burns in this area. An important conclusion that derives from this study, is the increase in mortality rate noted in the patients having perineal burns with an increase in the infectious potential. Advanced age, a high percentage of third-degree burns and a high ABSI score all represent negative prognosis factors that decrease the rate of survival. More research is essential to deepen our understanding of perineal burns and their implications for patient care. Proven to be a negative prognosis factor for burn patients, perineal area lesions should be treated rigorously, despite representing a small surface. Prevention of infections by adhering to a strict hygiene protocol, avoiding urinary and fecal contamination and removing debris and necrotic tissue is mandatory. A standardized treatment protocol depending on the burn depth should be implemented in any burn unit. Postoperative care and posturing must be carefully surveilled.

## Figures and Tables

**Table 1:** Characteristics of the study patients.

Variables		Category of Patients			Independence Test Chi-Square		
		Perineal (N = 49)	Non-Perineal (N = 198)	Total (N = 247)	χ2	df	*p* (2-Sided)
		19.8%	80.2%				
Sex	Female	13 (26.5%)	76 (36.4%)	89 (34.5%)	1.52	1	0.242
	Male	36 (73.5%)	133 (63.6%)	169 (65.5%)			
Age		60.96 ± 18.86	54.78 ± 17.59	t = −2.17		245	0.031
Range of age	≤40	6 (12.2%)	45 (22.7%)	51 (20.6%)	6.56	5	0.256
	41–50	10 (20.4%)	48 (24.2%)	58 (23.5%)			
	51–60	7 (14.3%)	33 (16.7%)	40 (16.2%)			
	61–70	6 (12.2%)	24 (12.1%)	30 (12.1%)			
	71–80	13 (26.5%)	29 (14.6%)	42 (17.0%)			
	>80	7 (14.3%)	19 (9.6%)	26 (10.5%)	Linear asoc		
Inhalation injury	Yes	34 (69.4%)	113 (57.1%)	147 (59.5%)	2.47	1	0.078
	No	15 (30.6%)	85 (42.9%)	100 (40.5%)			
%TBSA	≤60%	34 (69.4%)	170 (85.9%)	204 (82.6%)	7.41	1	0.011
	>60%	15 (30.6%)	28 (14.1%)	93 (17.4%)			
Mechanism	Chimic	1 (2.0%)	6 (3.1%)	7 (2.9%)	4.71	7	0.513
	Contact	1 (2.0%)	6 (3.1%)	7 (2.9%)			
	Electrical	7 (14.3%)	13 (6.6%)	20 (8.2%)			
	Explosion	9 (18.4%)	43 (21.9%)	52 (21.2%)			
	Flame	27 (55.1%)	104 (53.1%)	131 (53.5%)			
	Liquid	4 (8.2%)	19 (9.7%)	23 (9.4%)			
	Sun	0 (0.0%)	4 (2.0%)	4 (1.6%)			
	sequel	0 (0.0%)	1 (0.5%)	1 (0.4%)			
Degree	I–II	4 (8.2%)	75 (37.9%)	78 (32.0%)	15.94	1	0.001
	III	45 (91.8%)	123 (62.1%)	168 (68.0%)			
ABSI		10.88 ± 2.46	8.67 ± 2.70	t = −5.21		239	0.001
Status	Survival	11 (22.5%)	98 (49.5%)	109 (44.12%)	11.65	1	0.0082
	Deceased	38 (77.5%)	100 (50.5%)	138 (55.87%)			

## Data Availability

The dataset is available on request from the authors.
